# Semantic search using protein large language models detects class II microcins in bacterial genomes

**DOI:** 10.1128/msystems.01044-24

**Published:** 2024-09-18

**Authors:** Anastasiya V. Kulikova, Jennifer K. Parker, Bryan W. Davies, Claus O. Wilke

**Affiliations:** 1 Department of Integrative Biology, The University of Texas at Austin, Austin, Texas, USA; 2 Department of Molecular Biosciences, The University of Texas at Austin, Austin, Texas, USA; 3 John Ring LaMontagne Center for Infectious Diseases, The University of Texas at Austin, Austin, Texas, USA; Wageningen University, Wageningen, the Netherlands

**Keywords:** class II microcin, protein large language model, embedding

## Abstract

Class II microcins are antimicrobial peptides that have shown some potential as novel antibiotics. However, to date, only 10 class II microcins have been described, and the discovery of novel microcins has been hampered by their short length and high sequence divergence. Here, we ask if we can use numerical embeddings generated by protein large language models to detect microcins in bacterial genome assemblies and whether this method can outperform sequence-based methods such as BLAST. We find that embeddings detect known class II microcins much more reliably than does BLAST and that any two microcins tend to have a small distance in embedding space even though they typically are highly diverged at the sequence level. In data sets of *Escherichia coli*, *Klebsiella* spp., and *Enterobacter* spp. genomes, we further find novel putative microcins that were previously missed by sequence-based search methods.

**IMPORTANCE:**

Antibiotic resistance is becoming an increasingly serious problem in modern medicine, but the development pipeline for conventional antibiotics is not promising. Therefore, alternative approaches to combat bacterial infections are urgently needed. One such approach may be to employ naturally occurring antibacterial peptides produced by bacteria to kill competing bacteria. A promising class of such peptides are class II microcins. However, only a small number of class II microcins have been discovered to date, and the discovery of further such microcins has been hampered by their high sequence divergence and short length, which can cause sequence-based search methods to fail. Here, we demonstrate that a more robust method for microcin discovery can be built on the basis of a protein large language model, and we use this method to identify several putative novel class II microcins.

## INTRODUCTION

The increasing problems of antibiotic resistance, failure of first-line treatments for bacterial infections, and a limited pipeline of drugs under development suggest that novel approaches to fighting bacterial infections may be required (1, 2). One class of potential novel therapeutics is antibacterial proteins and peptides, which are found across all forms of life and are very diverse in structure and function (3, 4). Among these are class II microcins, a class of antibacterial peptides produced by gram-negative bacteria, which have shown some therapeutic potential *in vivo* (5, 6). Microcins are part of a larger group of antibacterial proteins, called bacteriocins, which are produced by bacteria and toxic to other bacteria. Mature class II microcins are less than 100 amino acids and 10 kDa, and their diversity and mechanisms of action are still largely unexplored (7). The larger, proteinaceous bacteriocins have recently become more popular as potential antibacterial therapeutics, in part due to rapid progress in protein sequencing and the expansion of bioinformatics data (8–10). Microcins also have the potential to be used as antibiotics, especially as more research on their structure and function becomes available.

Unfortunately, only 10 class II microcins with partial structural and/or functional characterization have been published to date. An early effort to screen for gram-negative bacterial peptides containing “double-glycine” signal sequences *in silico* may have identified microcins but was not explicitly designed to do so (11). More recently, our group designed a computational pipeline specifically for the detection of class II microcins (12). This pipeline, cinful, relies on both BLAST and a profile hidden Markov model (pHMM) trained on the 10 published class II microcin sequences (12). The application of cinful to over a thousand Enterobacterales genomes successfully expanded the sequence range of putative microcins. However, because of the limited number of experimentally verified microcins and the high sequence diversity among them, cinful may not work optimally for detecting the full range of novel, previously unidentified microcins that exist. Moreover, since microcins have high sequence divergence, there is a need for alternative approaches that do not primarily rely on alignments to detect microcins.

Here, we investigate an alternative method of identifying putative microcin sequences. Instead of employing sequence similarity as the primary search tool, we are instead searching for open reading frames (ORFs) that are close to known microcins in a high-dimensional embedding space generated by the protein large language model (LLM), ESM-1b (13, 14). First, we show that microcins cluster together in an embedding space when compared to non-microcin ORFs. Second, we show that almost every single one of the 10 known microcins can be used to recover all other known microcins from their genomic background using distance in embedding space. By contrast, BLAST searches perform poorly at the same task. Third, we use embeddings to search through *Escherichia coli*, *Klebsiella* spp., and *Enterobacter* spp. genomes where prior sequence-based methods found a microcin exporter gene but no actual microcins. In these genomes, our approach identifies novel putative microcin sequences.

## RESULTS

We wanted to know whether semantic embeddings from a protein LLM can be used to search for small proteins in bacterial genomes. Specifically, we were interested in detecting class II microcins, which are short (fewer than 150 amino acids) and highly diverged, making them difficult to find using traditional sequence-based approaches.

To assess the overall feasibility of our approach, we first asked whether embeddings for microcins cluster together when compared to embeddings for non-microcin ORFs. We generated embeddings for the 10 currently known microcins (File S1) using the protein large language model, ESM1-b (13). ESM1-b is a widely used LLM trained on 250 million known protein sequences. The complete set of ESM1-b embeddings for a single protein sequence of length L is a matrix of 1,280 rows and L columns. To reduce this data to a more manageable set of numbers, for each protein, we calculated the average across all L columns for each row and used the resulting set of 1,280 averages as our “semantic embedding” vector representing various characteristics of the biochemistry of the input protein.

**Fig 1:**
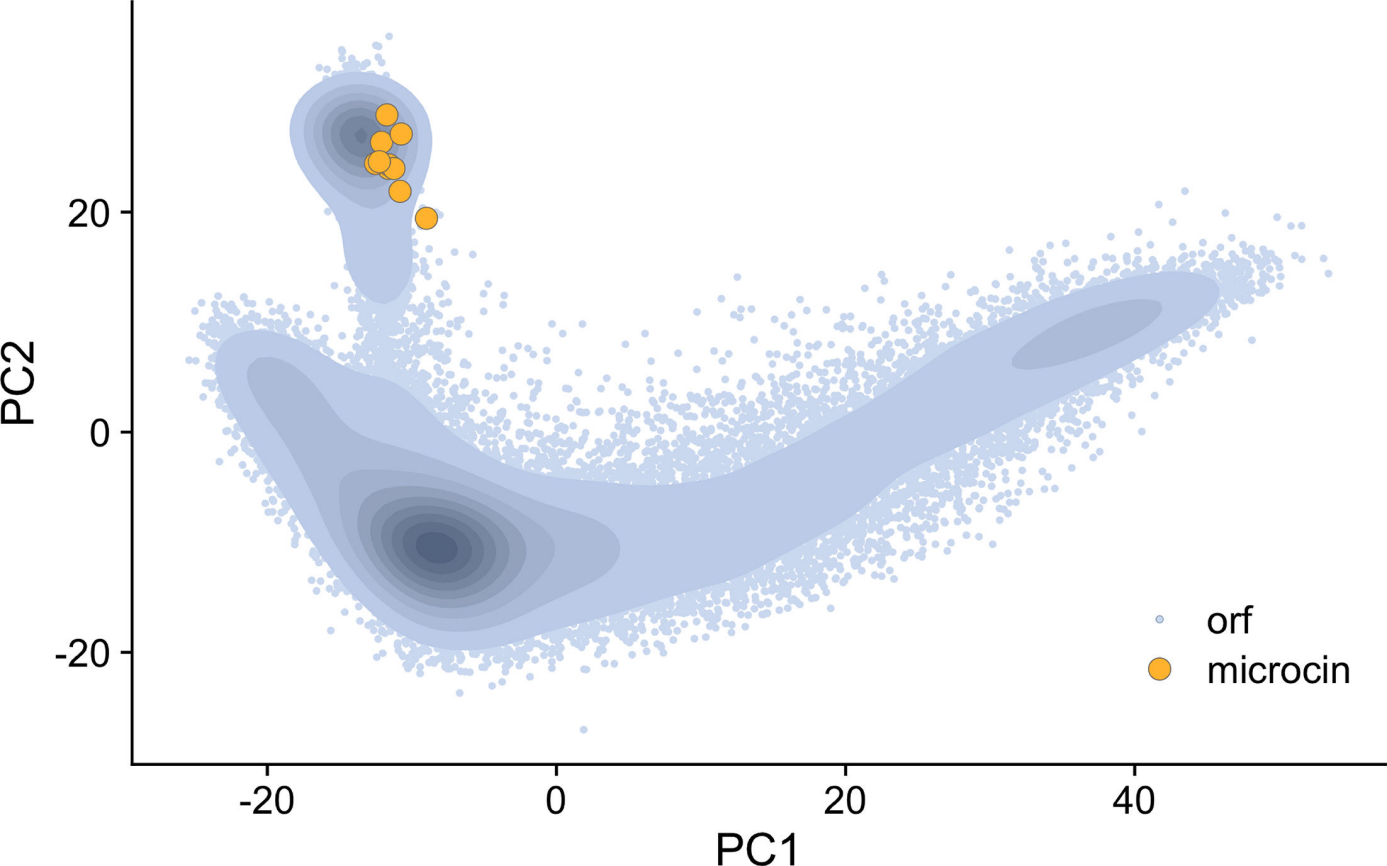
PCA plot of *E. coli* 54909 non-microcin ORFs and the 10 known microcins. Blue points represent non-microcin ORF embeddings, while the orange points represent embeddings of the 10 known microcins. Darker colors along the blue gradient show higher density of points for non-microcin ORFs. There are a total of 27,745 non-microcin ORFs.

For comparison, we similarly calculated semantic embedding vectors for all putative non-microcin ORFs of length 30–150 amino acids in the *E. coli* 54909 genome, for a total of 27,745 ORFs. We chose this genome as it is one of the two complete *E. coli* genomes in which microcin L is found. The large number of putative ORFs is due to our ORF extractor, which finds all contiguous stretches of non-stop codons beginning with ATG and within the correct size range (see Materials and Methods). This definition includes many low-confidence ORFs. For comparison, a common *E. coli* K-12 genome contains about 4,333 high-confidence ORFs. We then performed a principal component analysis (PCA) on the combined set of embeddings (microcin and non-microcin ORFs) and plotted the location of each ORF within the first two components (Fig. 1). We found that all microcins were tightly clustered within a small region of the overall embedding space, suggesting that microcins share similar characteristics that the embeddings can capture. However, non-microcin ORFs were similarly present in the same region of the first two principal components, i.e., the microcins did not trivially separate from the remainder of the ORFs in the genome. We emphasize that the full embedding space has 1,280 dimensions, so a more sophisticated approach considering all dimensions can potentially separate microcin ORFs from non-microcin ORFs even if they overlap in the two-dimensional PCA plot. More importantly, in the PCA plot, the microcin embeddings fell along the periphery of the ORF embedding distribution and did not overlap with the majority of non-microcin embeddings. Also, silhouette scores for the microcin group supported the notion that microcins form a coherent cluster in the embedding space (Fig. S1). The microcin group had a median score of 0.64 with very little dispersion around this value, indicating good cluster coherence. All other ORFs combined had a median score of 0.20, indicating poor clustering, but this was to be expected since these ORFs span a wide range of protein structures and functionality.

**Fig 2:**
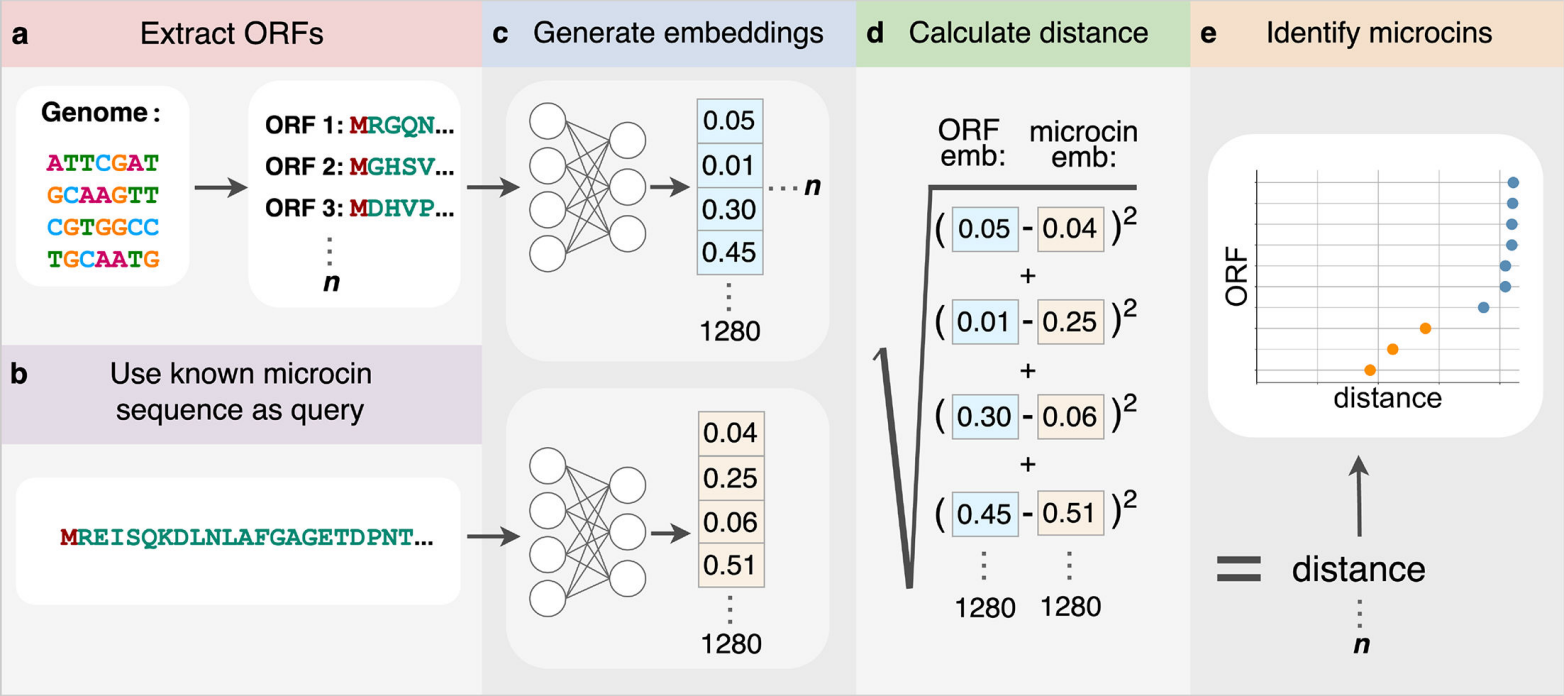
Detecting microcins using transformer embeddings. (**a**) ORFs are extracted from either whole genomes, gene clusters, or plasmids. (**b**) Each microcin sequence is consecutively selected as the query. (**c**) Embeddings are generated for both the extracted ORFs as well as the microcin query sequence. (**d**) Semantic distance is calculated as the Euclidean distance between the two embedding vectors. (**e**) Microcin ORFs (orange) are identified as a cluster of 1–3 points all having very small distances to the query microcin embedding.

**Fig 3:**
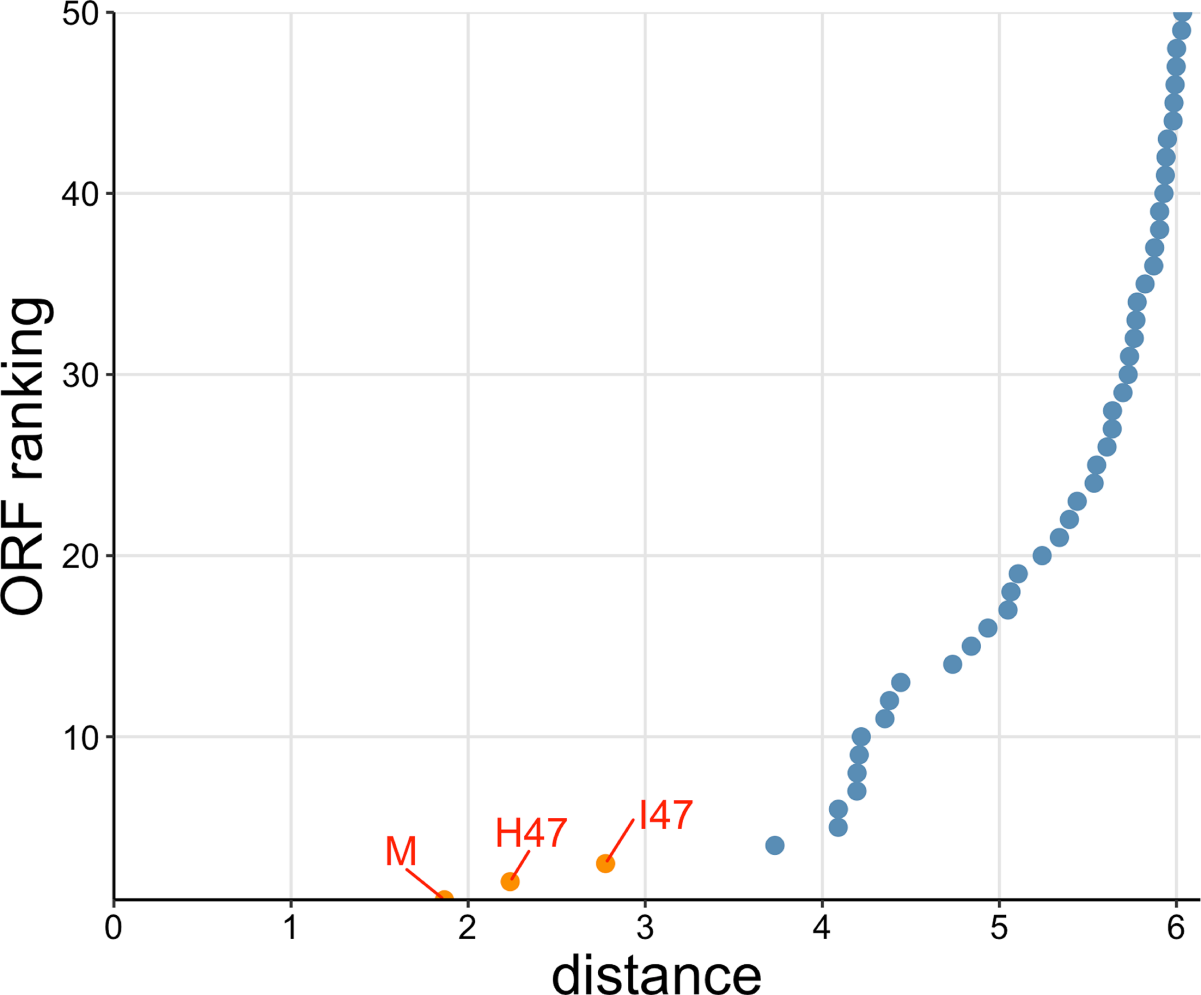
Search for microcins using microcin E492 as the query against the *E. coli* microcin H47 gene cluster. The microcin H47 gene cluster contains 405 extracted ORFs. Orange points indicate detected known microcins. Blue points indicate non-microcin ORFs. ORFs have been ranked based on increasing semantic distance.

Next, we tested whether or not each of the 10 known microcins can be used in a database search to find all other known microcins among a collection of ORFs extracted from either a genome or a gene cluster. We collected assemblies of genomes, gene clusters, or plasmids, as appropriate, which contain one or more of the known microcin sequences (Table 1). We extracted all ORFs for each of these assemblies (Fig. 2a). Because the known microcins range from 75 to 120 amino acid residues long, we discarded all ORFs that were either longer than 150 residues or shorter than 30 residues. We then generated ESM1-b embeddings for all extracted ORFs (Fig. 2c). Each of the 10 microcin embeddings was consecutively used as the query sequence to search within each assembly (Fig. 2b). As an additional query, we used the average embedding of the 10 microcin embeddings. We calculated the Euclidean distance between each ORF embedding in the assembly to the query microcin embedding and generated a set of distances for each query embedding (Fig. 2d). Finally, we plotted and ordered these distances within each set by ascending distance and only looked at the 50 smallest distances to the query (Fig. 2e). In total, each assembly produced 11 distance plots; 10 for the query microcins and 1 for the averaged microcin embedding. We expected that potential microcin ORFs would have the smallest distance to the query embeddings and appear separated from the rest of the non-microcin ORF embeddings (orange points in Figure 2e and 3 )

**TABLE 1:** Genomes, gene clusters, or plasmids containing the 10 known microcins^*a*^

Genome/cluster	Accession	Extracted ORFs	Microcins
*E. coli* pColV-K30	AJ223631.1	157	V
*E. coli* NCTC11128	GCF_900448705.1	25,819	N
*E. coli* 54909	GCF_025261725.1	27,746	L
*K. pneumoniae* cluster	AF063590.3	372	E492, G492
*E. coli* H47 cluster	AJ009631.3	405	I47, H47, M
*E. coli* G3/10 plasmid	JN887338.1	258	S
*E. coli* 25 plasmid	JQ901381.1	647	PDI

^*a*^ The column “extracted ORFs'” refers to the number of putative open reading frames remaining after filtering the initial extracted ORFs by size.

**Fig 4:**
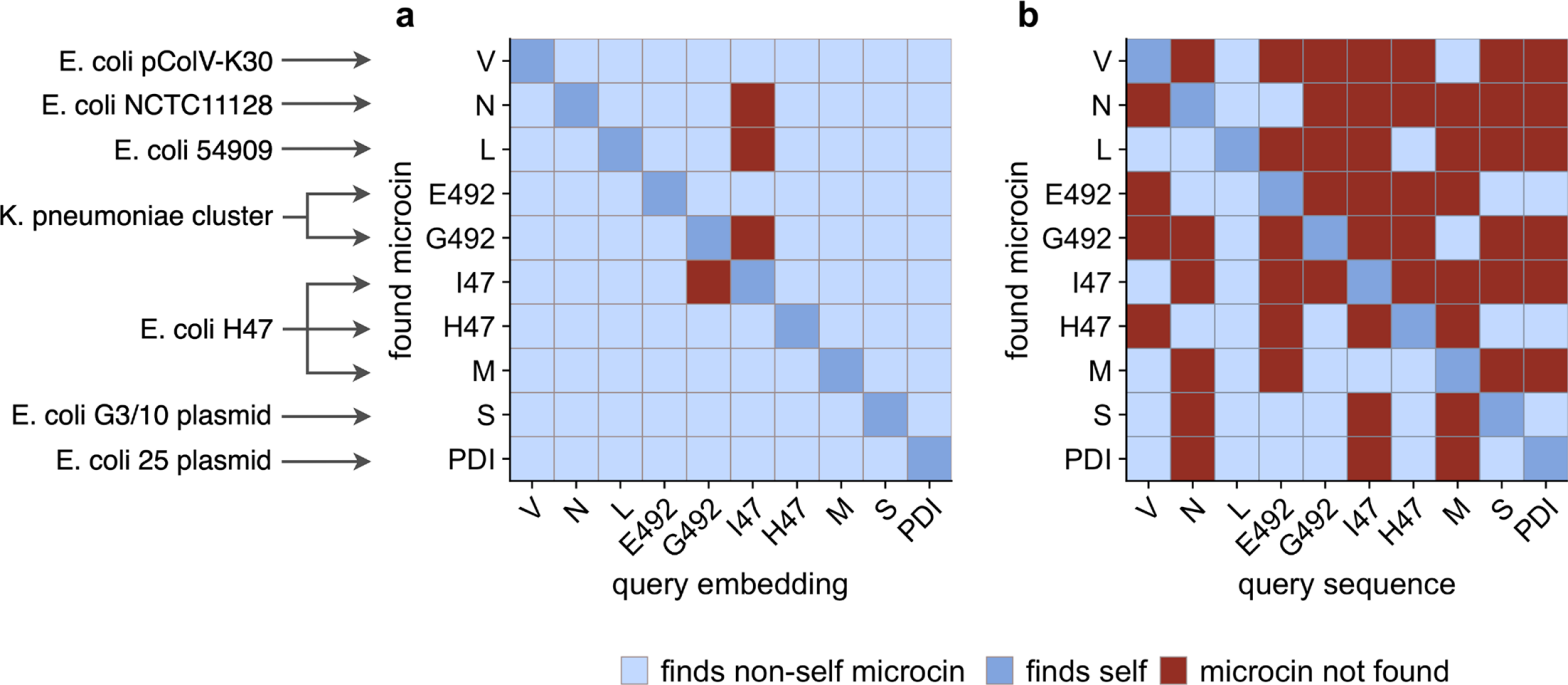
Performance of searches via semantic embeddings and via BLAST for known microcins across seven genomic assemblies. Light blue indicates that a microcin was found using a different microcin as the query. Dark blue indicates that a microcin was found using itself as the query. Red indicates that the microcin was not found. (**a**) When searching via semantic embeddings, nearly every individual known microcin can be found using any other known microcin as the query. (**b**) When searching via BLAST, most of the known microcins cannot be found using any other known microcins as the query. The only exception is the L microcin, which recovers all known microcins. BLAST parameters were as follows: *E*-value cutoff of 10 (default), alignment length ≥30 residues, and percent mismatch <80%.

To analyze the performance of this search algorithm more systematically, we defined a microcin as “found” if its distance was within the five lowest distances to the query (orange in Fig. 2e), and “not found” otherwise. We found that nearly every microcin query sequence was able to recover all of the 10 known microcins using this procedure (Fig. 4a). Furthermore, in most cases, the microcin ORFs were in the top two hits, even though we considered the top five in our procedure. The main exception was microcin I47 when used as a query, which did not recover microcins G492, N, or L. Also, microcin G492 when used as a query did not recover microcin I47. We note that microcins I47 and G492 had the largest distance in embedding space among all possible microcin pairings (Fig. S2). We also emphasize that the distance in embedding space between two microcins is not by itself the sole determining factor in whether a given query recovers a given target. Microcins I47 and H47 had the second-largest distance in embedding space (Fig. S2), and either one could be used to recover the other (Fig. 4a). Whether a known microcin is recovered within the five lowest distances depends not only on the distance from the query to that microcin but also on whether there are any other ORFs that happen to have a low distance in embedding space, regardless of whether or not they are potential microcins. In fact, in general, it was more challenging to identify microcins (such as N and L) located in complete genomes with thousands of extracted ORFs rather than in small gene clusters with only hundreds of ORFs (see Table 1 for the exact ORF numbers used in this study). Yet, all microcins other than I47 when used as the query were able to recover both microcins N and L.

To assess whether our results were influenced by the choice of distance metric, we considered two additional distance functions: the cosine distance and the Manhattan distance. We found that the cosine distance performed poorly for the two most challenging cases, the N and L microcins. These two microcins could not be recovered from any of the other microcins using the cosine distance (Fig. S3). By contrast, the Manhattan distance performed even better than the Euclidean distance, which already performed very well. Using the Manhattan distance, the I47 microcin embedding was able to also find the N microcin, and the G492 embedding was able to also find the I47 microcin (Fig. S4).

We contrasted our embedding-based search to a standard BLAST search. We took the same extracted ORFs we had used for the embedding method and employed them as the database sequences for BLAST. We then ran BLAST searches with each of the 10 microcin sequences as a query. We used an *E*-value cutoff of 10 and imposed no restrictions on the alignment length or percent mismatch. Nevertheless, despite using these extremely lenient search criteria, very few microcin query sequences were able to recover the majority of the other microcin sequences. In fact, most microcins only recovered two to three other microcins. And many of the BLAST hits were of very poor quality and would normally be filtered out, such as hits with *E*-values >0.1 or very short alignment lengths <20 (File S2).

The one microcin that worked reasonably well as a query sequence for BLAST was microcin L, which was able to recover all other microcins (Fig. 4b). It is unclear why microcin L performs so much better than the other microcins. In terms of sequence divergence, it shows similar values to any other microcin pairs. Nearly all known microcins are between 50% and 70% diverged from any other microcins (Fig. S5).

We also wondered whether a more simple measure, amino acid composition, could be used instead of semantic embeddings to identify microcins. Since microcins are short and enriched in glycines, it is conceivable that amino acid composition alone is sufficient to recognize them. However, using Euclidean distance between amino acid composition vectors as our metric for sequence similarity, we found that amino acid composition was not sufficient to reliably identify microcins, even though it did perform somewhat better than BLAST (Fig. S6).

The quality of a BLAST hit is commonly represented by its *E*-value, which is defined as the number of expected hits of similar quality that could be found just by chance. We can define a similar quantity for our distance-based search, by recognizing that under the assumption of random sampling, the number of microcin hits among the top five shortest distances follows a hypergeometric distribution. The expected value of the hypergeometric distribution is *nK*/*N*, where *n* is the number of draws (here, *n* = 5), *K* is the number of true microcins in the data set, and *N* is the total number of ORFs. Using this formula with the data from Table 1, we find that the largest expected number of microcin hits among the top five distances under random sampling is 0.037 (for *E. coli* H47, which has 405 ORFs and 3 microcins). In all other cases, the expected number of hits is even lower. For example, for *E. coli* 54909, we have 5/27,746 = 0.0002. Similarly, instead of calculating expected hits, we could calculate *P*-values using the hypergeometric distribution, by calculating the probability of finding at least one hit in the top five under the assumption of random sampling, and we obtain *P*-values that are numerically in a similar range as the expected values. (The formula *nK*/*N* is a good approximation for the tail probability when *nk* ≪ *N*.)

**Fig 5:**
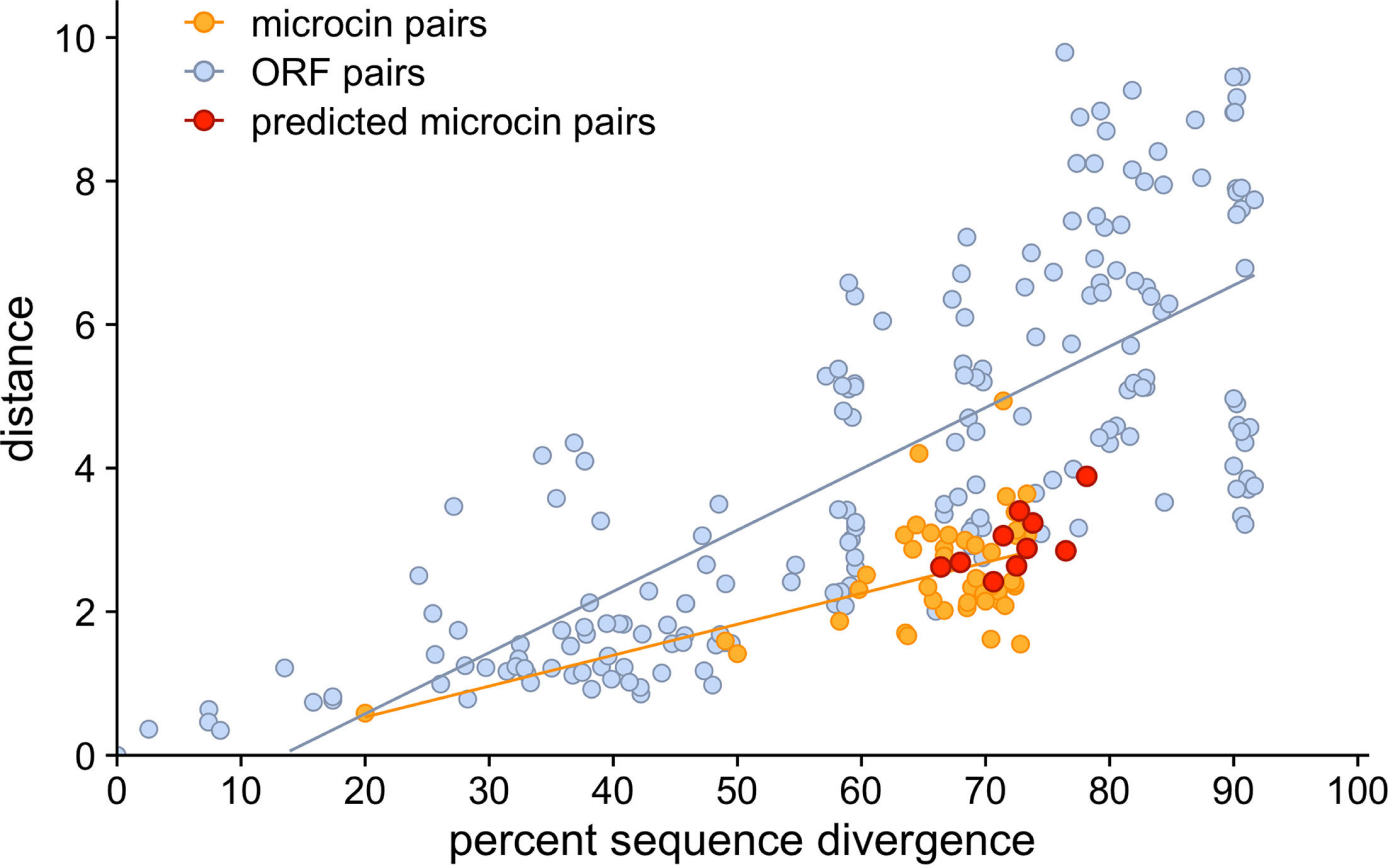
Semantic distance and percent sequence divergence for microcin pairs and non-microcin ORFs of comparable length. Blue dots indicate non-microcin ORF pairs, while orange dots indicate pairs of known pairs. For non-microcin ORF pairs, there are 194 pairs sampled across the range of sequence similarities. The microcin pairs include all 45 pairings of each of the 10 known microcins with every other one. The predicted microcin pairs include 10 pairings between each of the 10 known microcins and the putative microcin from *E. coli* TA097 identified in this work (see also Fig. 6).

BLAST has difficulty finding microcins because they are both short and highly diverged from each other. By contrast, distances in embedding space are small even for highly diverged microcins. To further explore the relationship between sequence divergence and distance in embedding space, we compared these distances for randomly chosen ORF pairs from the *E. coli* 54909 genome. Specifically, we picked 194 pairs of non-microcin ORFs (sampled at random such that they covered the entire range of sequence divergence values) and compared them to all possible microcin–microcin pairs. We found that, compared to non-microcin ORF pairs, microcins tended to have low semantic distance between each other given their sequence divergence, which was around 70% on average (Fig. 5). Thus, in other words, even though microcins are highly diverged, they possess unique characteristics that can be picked up by the embedding space of a large language model, and they can be uniquely identified by these characteristics.

Having established that embeddings may outperform sequence-based methods to locate microcins, we next asked whether we could discover novel putative microcins. In a previous study (12), we used the software cinful, a sequence-based method for microcin discovery, to search for putative microcins in the Touchon data set of ~1,000 *E. coli* genomes (15). From this large data set, we compiled a smaller data set of 25 genomes where cinful had not previously found any putative microcins but did find the peptidase-containing ABC transporter (PCAT) microcin exporter gene (File S3). We reasoned that any genomes containing a microcin exporter would be prime candidates to contain microcins that may have been overlooked using the earlier methods.

For this systematic search for novel putative microcins, we proceeded as before, first calculating embeddings for all ORFs, then calculating distances between these embeddings and the embeddings of known microcins, and then looking for the ORFs with the smallest distances to known microcins. However, we slightly modified our definition of a hit to be more restrictive. We now only considered ORFs to be putative microcins if they were among the lowest two distances in the distribution of ORF distances for more than two query sequences and if there was also a meaningful gap between these distances and the rest of the distance distribution.

Furthermore, prior to analyzing any putative novel microcin hits, we needed to set criteria for what we would consider to be a likely true positive. Class II microcins have several characteristics that distinguish them from non-microcin peptides (7). First, microcins contain a short (15–18 amino acid) “double-glycine” signal sequence at the N-terminus. This signal sequence terminates in either a glycine–glycine (GG) or glycine–alanine (GA) residue pair. Double-glycine signal sequences typically contain hydrophobic residues at positions −4, −7, and −12 relative to the signal-sequence cleavage site. These hydrophobic residues are critical for the efficient secretion of double-glycine signal-containing peptides (16). Class II microcin sequences are also unusually rich in glycine residues compared to other proteins, with glycine content ranging from 12% to 26%. Finally, class II microcins are encoded near their PCAT export gene on the genome. All of these characteristics were taken into account when identifying putative microcins among the output from the embedding search.

**Fig 6:**
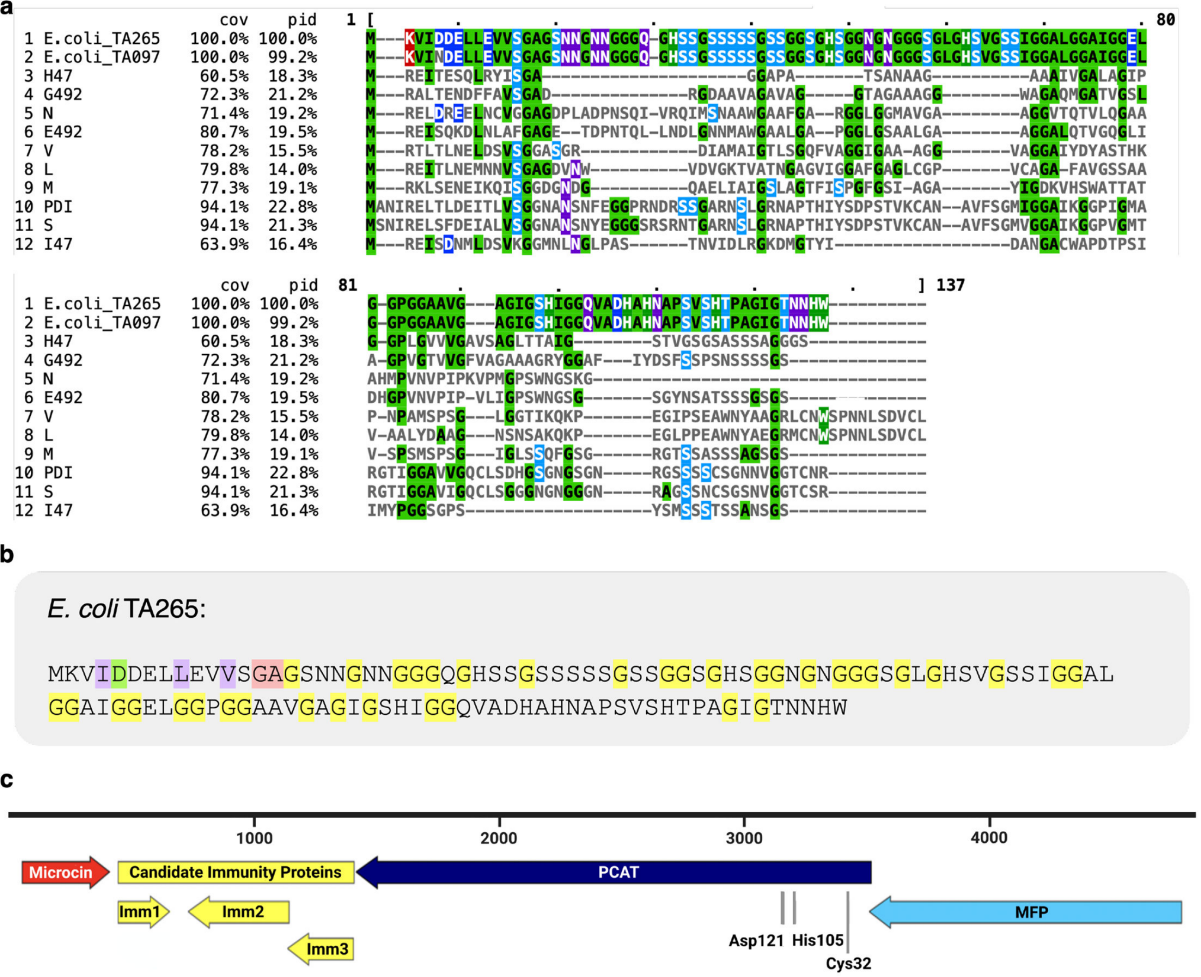
Putative microcins detected within 25 *E. coli* genomes. (**a**) Alignment of two putative microcins from *E. coli* TA265 and TA097 to the 10 known microcins. “pid” represents percent identity to microcin TA265. (**b**) Putative microcin sequence TA265. There is a single residue difference between the two found homologs; the other homolog (TA097) contained asparagine (N) instead of aspartic acid (D) at the position highlighted in green. The two residues before the signal sequence cleavage site are shown in pink. Hydrophobic amino acids (purple) are found at positions −4, −7, and −12 relative to the cleavage site at the end of the signal sequence. An abundance of glycine (yellow) is found throughout the sequence. (**c**) Genomic context of the putative microcin from *E. coli* TA265 (4,835 bp). A PCAT and membrane fusion protein (MFP) necessary for microcin export are encoded near the putative microcin. The PCAT contains the critical peptidase domain residues (Cys32, His105, and Asp121) necessary to cleave the signal sequence of the putative microcin during export (18, 19). Putative small proteins (70–136 amino acids) with predicted transmembrane domains are located between the microcin and PCAT; these are candidate immunity proteins.

Among the 25 genomes with a PCAT microcin exporter gene but no previously discovered putative microcins, our embedding method detected a total of 44 hits (File S4). Out of the 44 hits, three were identified as putative microcins (Table 2; Fig. 6; File S5). One of those, microcin L, located within the 36_1_Ti13 genome, had previously been missed because it had not been identified as an ORF by cinful’s ORF finder, Prodigal (17). The more inclusive definition of ORFs we used here allowed us to find this microcin. The two other hits, however, were novel microcin-like sequences, discovered in the two genomes TA265 and TA097, respectively (File S5; Fig. 6a). Both of these sequences displayed characteristics of microcins, such as high glycine content (yellow), a glycine–alanine (GA) residue pair before the cleavage site (pink), and hydrophobic residues (purple) at positions −4, −7, and −12 relative to the signal sequence cleavage site (Fig. 6b). We also found that these two new putative microcins had similar sequence divergence and semantic distance to known microcins (Fig. 5). We confirmed that these sequences had been identified as ORFs in the cinful pipeline and had been missed by the BLAST search employed by cinful.

**TABLE 2:** Results from *E. coli*, *Enterobacter*, and *Klebsiella* genomes^*a*^

Data set	Genomes	Total hits	Unique hits	Unique microcins
*E. coli*	25	44	20	3
*Enterobacter*	44	63	26	9
*Klebsiella*	46	74	19	16

^*a*^ In all three sets of genomes, no microcins have previously been detected, while the PCAT microcin exporter gene has been found. The column “unique microcins” refers to the number of unique putative microcins found.

Using the microcin-like sequence discovered in *E. coli* TA265 as an example, we examined the genomic region surrounding the putative microcin to determine if genes encoding the other expected proteins necessary for export were present (Fig. 6c). Indeed, both the PCAT and membrane fusion protein (MFP are encoded near the putative microcin. We further confirmed the PCAT contains the three critical peptidase domain residues necessary for the cleavage of the signal sequence during export (18, 19); this functionality differentiates the unique microcin type I secretion system from all other type I secretion systems (20). We also found three putative small proteins encoded between the microcin and the PCAT, which are candidate immunity proteins. Using DeepTMHMM (21), we found that they are all predicted to have two or more transmembrane domains, which is a feature of known microcin immunity proteins. Collectively, all genomic context for the putative microcin from *E. coli* TA265 is consistent with features expected for a microcin (7).

Next, we used the same method to scan 44 *Enterobacter* spp. and 46 *Klebsiella* spp. genomes from GTDB for potential novel microcins (12). These two datasets also contained a previously detected PCAT microcin exporter gene but had no detected putative microcins. In total, out of the 44 *Enterobacter* spp. genomes, there were 63 total hits that contained 26 unique sequences (Table 2; File S6). Within these hits, nine had features indicative of class II microcins; they contained a double-glycine signal sequence and had high glycine content and characteristic hydrophobic residues upstream of the signal sequence (File S5). Out of the 46 *Klebsiella* spp. genomes, there were 74 total hits containing a total of 19 unique sequences that we identified as putative microcins (File S7). Sixteen of these hits displayed microcin-like features (File S5). However, four of these had a glycine content in the range of 8%–10%, which is low compared to known microcins (File S1). The other 12 *Klebsiella* spp. sequences had a glycine content of 2%–26% and a characteristic double-glycine signal sequence. It is important to note that, while the rate at which microcins were found is good for *Klebsiella* spp. (16/19), only 9/26 *Enterobacter* spp. hits appeared to be true microcins. The reason for this could be that the query embeddings are generated only from the 10 known microcins, eight of which are from *E. coli*, two of which are from *Klebsiella pneumoniae*, and none of which are from *Enterobacter* spp. Therefore, this confirms the need to assess antibacterial activity from newly discovered microcins so that a wider diversity of query embeddings can be used in future studies.

Finally, we looked at 40 additional *E. coli* genomes including 20 from isolates from water and 20 from human extra-intestinal isolates from the B2 phylogroup (15) (File S8). We chose these particular two genome groups because our prior analysis using cinful identified them as having the least and most class II microcin hits, respectively (12). Fifteen of the genomes from water-isolated *E. coli* had no previously detected microcins, and 10 of the genomes from human extra-intestinal-isolated *E. coli* had no previously detected microcins.

Among the 20 water-sourced genomes, cinful detected a total of eight putative microcins, and our embedding method detected nine: one additional instance of microcin H47 was found by the embeddings and missed by the pHMM step in cinful. For the human extra-intestinal genomes, cinful detected 10 putative microcins and the embedding method found 12. The additional two microcins found by the embedding method were also microcins H47 and M, previously missed by the pHMM step of cinful. In summary, out of all 40 genomes analyzed, 18 microcins were found by cinful, and 21 were found by the embedding method. We emphasize, however, that the three additional microcins found by the embedding method were identical at the sequence level to previously known microcins yet were missed by the pHMM.

## DISCUSSION

We have assessed the ability of protein large language models, and specifically the embeddings produced by such models, to detect class II microcins in bacterial genomes. We have found that a simple method based on distance in embedding space has much higher sensitivity to discover highly diverged and relatively short microcin sequences than do sequence-based methods such as BLAST. We have applied our method to a subset of previously analyzed *E. coli*, *Enterobacter* spp., and *Klebsiella* spp. genomes and have discovered both known and novel putative microcin sequences that prior sequence-based methods have missed.

Protein LLMs have seen massive success in recent years in predicting the biochemical, structural, and sequence properties of proteins (22). One of the most widely used classes of such models is the ESM family of protein language models. These models have been trained on millions of protein sequences and can be used to predict the biological structure and function from sequence data (13, 14, 23). At the core of all protein large language models are so-called embeddings, which encode protein characteristics into a high-dimensional numerical space suitable for many different downstream applications. In particular, recent works have shown that embeddings can be used for the identification of protein homologs, even in cases of low sequence similarity (24–27).

Sequence-based genomic search methods have been the standard for identifying homologous protein sequences. Most of these methods are based on global or local sequence alignments. A critical limitation of sequence-based methods is that they rely on significant sequence similarity between the query and the detected homolog sequence. However, homologs may be diverged in sequence while retaining their overall structure and function (24). Such is the case with class II microcins; they are a unique group of proteins that are both very small and have high sequence divergence relative to each other.

The short length and high sequence divergence of class II microcins create problems for sequence-based methods but provide advantages for the embedding method. First, the short sequence length reduces the computational cost of generating sequence embeddings; since the attention mechanism in large language models scales quadratically with the length of the input sequence, the shorter the sequence, the faster the embeddings can be calculated. Second, the high sequence divergence makes it particularly problematic to generate accurate alignments, whereas embeddings seem to be much less affected by sequence divergence. In part, this is likely due to the fact that embeddings provide a global perspective on the entire sequence whereas BLAST looks for high-quality local matches. With sufficient sequence divergence, there may not be any local region in a target sequence that appears sufficiently similar to the query, even if from a global perspective the two sequences share meaningful homology.

In terms of computational cost, the embedding-based method can be more costly, but exact details depend on how large of a sequence database needs to be searched and how many times. The main cost is the initial computation of embeddings for every sequence to be searched against, whereas the distance computations are extremely rapid. Therefore, repeated searching of the same database for different queries will be fast. Moreover, the computation of embeddings can be slow on a CPU but will be very fast on a state-of-the-art GPU such as an Nvidia H100. In practice, assuming access to a performant GPU, the embedding-based approach can be scaled to similar data set sizes as using BLAST.

The raw embeddings produced by ESM are very high-dimensional, as they consist of a 1,280-dimensional feature vector for each position in the input sequence, corresponding to a total of 1,280 × *L* values for a protein of length *L*. For any downstream analysis, it is necessary to reduce the dimensionality of this feature space. We did this here by taking the average across all sites, retaining 1,280 mean values for each peptide. This averaging across sites is a commonly employed strategy in the field of protein language models (26–29). Because the embeddings are generated by an iterated application of the transformer attention architecture, which considers each position in the context of all other positions in the sequence, the final embeddings are expected to contain global information about the input sequence, and averaging along the length dimension makes sense. In fact, our results here show that mean embeddings work well to differentiate microcins from other peptide sequences. We also note that the mean embeddings contain information beyond just the amino acid composition of the input sequence, as amino acid compositions did not perform nearly as well in recovering microcins, in particular for searches across larger genomes containing many thousands of ORFs.

The known microcins have several characteristics that distinguish them from non-microcin peptides, including specific features of their signal sequences, glycine content, and genomic location near their PCAT exporter (7). Our embedding method recovered novel sequences that unequivocally displayed these characteristics (Table 2). Additionally, however, we also found hits that do not fully look like canonical microcins (File S1). In particular, some of our detected hits had only around 8%–10% glycine content yet displayed other microcin-like features such as a double-glycine signal, appropriate hydrophobic residues upstream of the signal, and a microcin-like size (<150 residues). These sequences are interesting candidates for future experimental validation.

We would like to emphasize that some of the hits we identified did not appear to have microcin-like features and are likely false positives. Many of these false positives did have a high glycine content, which may explain why they were found by the embeddings. However, they lacked other microcin features, such as a double-glycine signal sequence. As mentioned above, one reason why false positives may be identified is that we are using averaged embeddings, and certain important protein features may be lost during the averaging step. A second reason for false positives is that our method for identifying microcin hits relies on a simple distance metric and distance cutoff; therefore, we can expect to occasionally find sequences that are not microcins but may share some characteristics with real microcins. These peptides could have either lost some of their microcin properties over evolutionary time or by coincidence just have microcin-like features such as high glycine content. Although we found apparent non-microcin hits, our method has very good sensitivity and does not appear to miss microcins when they are present in the genome.

Our work has also revealed that our prior tool for microcin search, cinful (12), can miss both known microcins and novel putative microcin sequences. We found candidate sequences to be lost at all three stages of cinful: the ORF extraction stage via Prodigal (17), the BLAST stage, and the pHMM stage. Because here we used an extremely broad definition of ORF, considering all sequences in the microcin length range starting with a start codon and ending with a stop codon, our ORF extraction step was unlikely to overlook any potential microcins. It is worth noting that we did not use alternative start codons because it would have vastly inflated the number of extracted ORFs for each assembly. However, since alternative start codons are rare, we believe this causes minimal problems in detecting microcins. Next, as shown here, BLAST tends to have difficulty identifying microcins because they are short and highly diverged. In addition, BLAST tends to generate false positives, i.e., it recovers sequences that are not microcins. This propensity of BLAST to generate false positives required the addition of a final filtering stage, the pHMM stage, in cinful. But generating a good pHMM was hindered by the small number of known microcins (12). As more microcins get discovered and verified, it is likely that both BLAST and pHMM searches can become more accurate, and an updated future version of cinful with a larger set of reference microcin sequences may perform better.

Our ORF extraction approach was extremely exhaustive, thus minimizing the chance of missed hits. However, this approach comes with the drawback of substantially increased computational cost, as all the putative ORFs need to be processed, and embedding calculations are not cheap. We believe that in the future, a more targeted search strategy may be appropriate and sufficient. Microcins tend to co-locate with their (highly conserved) PCAT and (MFP, which are used for their export. Because PCATs and MFPs are so conserved, they are easier to identify in a sequence-based search than microcins (7, 12). Therefore, future searches could restrict the search space only to ORFs located within close proximity to PCAT or MFP sequences. Alternatively, or in addition, one could use distances to biosynthetic clusters, such as BiG-SCAPE distances (30), to assess the likelihood that a given candidate sequence is a genuine microcin.

There are of course other methods of searching for remote homologs beyond embeddings on the one hand and BLAST on the other. First, there are hidden Markov models (HMMs) that build sequence profiles of either the query sequence (31) or the query and the target sequences (32, 33) and perform searches based on these sequence profiles. HMMs work the better the larger the sequence alignments from which sequence profiles are built. Our prior work on cinful (12) has shown that it is possible to build sequence profiles to represent microcins and search based on those profiles. We see the embedding approach as complementary. It can be a useful tool either to independently confirm results obtained with HMMs or to apply when so few query sequences are available that sequence profiles cannot be constructed. Second, it is possible to perform searches based on protein structures, for example, by folding all sequences with AlphaFold (34) or taking advantage of a pre-calculated AlphaFold database (35, 36). However, for shorter peptides, computational structure prediction tends to be unreliable, and therefore, it is not a feasible strategy for the microcins we have considered here.

In summary, our work has shown that protein embeddings produced by large language models can be used successfully to search for small, highly diverged sequences such as class II microcins in genome-scale data sets. Embeddings have several advantages over sequence-based methods and appear to have both higher sensitivity and higher specificity for detecting novel microcins. However, embeddings can incur higher computational costs than a BLAST search or a hidden Markov model and, therefore, may be most suitable for applications where sequence-based methods clearly fail or where the search space is somewhat limited.

## MATERIALS AND METHODS

### Data collection

We collected seven genomes, plasmids, or gene clusters (Table 1) that contained the 10 known microcins. These microcins are class IIa microcins V (37), L (38), N (39), PDI (40), and S (41) and class IIb microcins H47 (42), I47 (43), M (44), E492 (45), and G492 (46). The genomic sequences containing these microcins were used to validate embedding distance as a tool to search for microcin sequences in genomic data.

To search for novel class II microcins, we compiled three datasets containing 25 *E. coli*, 44 *Enterobacter* spp., and 46 *Klebsiella* spp. genomes, respectively (File S3). When choosing the genomes for each data set, we looked at genomes where the previous method, cinful, did not find any microcins yet found the PCAT microcin exporter gene (12). These genome data sets were taken from a subset of the GTBD genomes that were used in reference (12). We next extracted ORFs from each genome, generated embeddings for each ORF, and searched each of the 25 genomes using the embeddings from each of the 10 known microcin sequences.

We also searched through 40 *E. coli* genomes from the B2 phylogroup from reference (15) (File S8). Of these 40 genomes, 20 were from *E. coli* isolated from water samples, 15 of which had no previously detected microcins (12). The other five were the only genomes from water isolates where microcins had been found by cinful (12). The other 20 genomes were from *E. coli* isolated from human extra-intestinal samples; we selected 10 genomes where putative microcins had been found and 10 genomes where no microcins had previously been found (12).

### Extracting ORFs and generating embeddings

We used a simple method to identify putative ORFs in each genome or gene assembly: we identified all contiguous stretches of codons starting with ATG (methionine) and ending with one of the three stop codons. We further imposed a minimum size limit of 30 residues and a maximum size limit of 150 residues, since all known microcins fall within these limits. In cases where ORFs had multiple possible start codons, we only kept the longest ORF within the size limit stated above. This process will recover many putative ORFs that do not correspond to known protein-coding genes. We accepted this high false-positive rate in our ORF-finding procedure since our goal was to detect as many putative microcins as possible, and some of them might be missed under a more restrictive definition of an ORF. For each genome, we searched for ORFs in all possible reading frames starting from methionine, both on the + and the − strand.

We generated ESM1-b embeddings for each ORF using the ESM library available at https://github.com/facebookresearch/ESM. Embeddings were initially generated for each residue in a sequence. Each embedding consists of a numeric vector of 1,280 features. To arrive at a single embedding per ORF, we subsequently averaged each embedding feature (one of the 1,280 numbers) across all residues in the sequence. The resulting averaged embedding is a numeric vector of 1,280 values representing the entire ORF. We generated these averaged embeddings for all extracted ORFs and all known microcins.

### Principal component analysis

For the PCA, we used 27,745 putative ORFs of microcin length (30–150 amino acids) from the *E. coli* 54909 genome. The ORFs were extracted and converted into embeddings as described above. The *E. coli* 54909 genome contains the known microcin L, which we removed from the putative ORFs to arrive at the non-microcin ORF group. For the microcin group, we used the embeddings for the 10 known microcins.

Silhouette scores were calculated for the microcin and non-microcin groups in python using the function silhouette_samples() from the sklearn.metrics library.

### Calculating embedding distance and identifying microcin hits

The mean protein embeddings are vectors of *n* = 1,280 features. To assess the similarity between embedding vectors, we calculated the distances between them. Specifically, assuming two embedding vectors *p_i_* and *q_i_* (with *i* = 1, 2,...,1,280), we calculated the Euclidean distance:


(1) $$d_{\text{Euclid}}=\sqrt{\sum_{i}(p_{i}-q_{i}{)}^{2}}.$$

We also considered two alternative distance metrics, cosine distance:


(2) $$d_{\text{cosine}}=\frac{\sum_{i}p_{i}q_{i}}{\sqrt{\sum_{i}p_{i}^{2}}\sqrt{\sum_{i}q_{i}^{2}}}$$

and Manhattan distance:


(3) $$d_{\text{Manhattan}}=\sum_{i}|p_{i}-q_{i}|.$$

All results in the main body of the text were obtained using Euclidean distances between the embeddings. The results for cosine and Manhattan distances are reported in supplemental figures.

After calculating the distances between a query microcin embedding and the embedding for each ORF in the target genome, we plotted ORFs in the order of ascending embedding distance and manually inspected the lowest 50 distances. For each target genome, we repeated this process 11 times, once for each of the 10 known microcins and once for the average embedding of all 10 microcins. Most genomes will generally contain one to two microcins, and it is rare for an assembly to have more than two microcins (12). For this reason, we inspected the two ORFs with the lowest embedding distance to any of the queries in each genome. If any one or two ORFs displayed a meaningful gap from the remainder of the distribution of distances and if they were found to be one of the two lowest distance ORFs by more than one query microcin, these one/two ORFs were declared “hits” and further inspected for microcin features.

Multiple sequence alignments of microcin and putative microcin sequences were performed using MAFFT from the EMBL-EBI search and sequence analysis tools (47), available at https://www.ebi.ac.uk/jdispatcher/msa/mafft. Alignments were visualized using MView from the EMBL-EBI search and sequence analysis tools, available at https://www.ebi.ac.uk/jdispatcher/msa/mview.

### Identifying microcins based on amino acid composition

To assess whether amino acid composition was sufficient to identify microcins, we used amino acid composition vectors instead of embedding vectors and repeated the analysis described in the preceding subsection. In brief, we calculated composition vectors as the 20 numbers corresponding to the relative proportion of each amino acid in an ORF or microcin sequence. We then calculated Euclidean distances between composition vectors and ranked results by the smallest distance, as before.

### Running BLAST

To run BLAST searches, we first took all extracted ORFs from the respective genome or gene assembly to be searched and converted them into a BLAST database. We then used each of the 10 known microcin sequences as a query against each of these databases.

All queries were run with protein BLAST v2.13.0 using an *E*-value cutoff of 10 and a maximum of 50 target sequences. We then checked all returned BLAST hits to see if they matched or overlapped with known microcin sequences.

### Calculating percent sequence divergence

Percent sequence divergence was calculated by first aligning a pair of sequences and then counting the total number of sites that did not match and dividing by the length of the alignment. Any gaps in the alignment were also treated as mismatches. All pairwise alignments were performed using gemmi (v0.5.7) (48).

To analyze sequence divergence and embedding distance of *E. coli* ORFs, we used the ORFs extracted from the *E. coli* 54909 genome containing microcin L. We first calculated sequence divergence for 112,017,666 arbitrarily selected non-microcin ORF pairs, which corresponds to approximately 15% of all total possible pairs. The initial sample was chosen by systematically calculating sequence divergence for all possible ORF pairs until we had collected a large number of pairs spanning a wide range of sequence divergence. We then further downsampled this data set for visualization purposes. The goal of our downsampling procedure was to obtain a uniform representation of the entire range of possible sequence divergence values. To this end, we binned all data into bins spanning 10 percentage points in sequence divergence. Because low-divergence ORF pairs are rare, we kept all data within the lowest three divergence bins from 0% to 30% divergence. Next, since the 30%–40% divergence group only had 25 pairs, we kept those pairs and arbitrarily selected 25 pairs for the remaining higher divergence bins. In the final sample, there were a total of 194 pairs; 175 pairs between 100% and 30% divergence (25 per 10% bin), nine pairs with divergence between 20% and 30%, four pairs between 10% and 20%, and six pairs between 0% and 10%. After downsampling, we generated embeddings for all ORFs in the pared data set and calculated the embedding distance for all ORF pairs. We also calculated sequence divergence and embedding distance for all possible combinations of the 10 known microcins, resulting in data for 45 pairs.

### Predicting transmembrane helices on putative immunity proteins

We used DeepTMHMM (21) to predict transmembrane helices on putative immunity proteins. The microcin genome map was generated using SnapGene software (https://www.snapgene.com/).

## Data Availability

All analysis scripts and processed data are available on GitHub: https://github.com/akulikova64/microcin_embeddings_project.

## SUPPLEMENTAL MATERIAL

File S1 msystems.01044-24-s0001.txt Amino acid sequences of the 10 known microcins, provided in FASTA format.

File S2 msystems.01044-24-s0002.csv Microcin--microcin BLAST hits.

File S3 msystems.01044-24-s0003.xlsx Information about the *E. coli*, *Enterobacter*, and *Klebsiella* genomes.

File S4 msystems.01044-24-s0004.pdf Alignment of embedding hits collected from 25 *E. coli* genomes.

File S5 msystems.01044-24-s0005.txt Putative microcins found in the *E. coli*, *Enterobacter*, and *Klebsiella* data sets.

File S6 msystems.01044-24-s0006.pdf Alignment of embedding hits collected from 44 *Enterobacter* genomes.

File S7 msystems.01044-24-s0007.pdf Alignment of embedding hits collected from 46 *Klebsiella* genomes.

File S8 msystems.01044-24-s0008.csv Cinful results for 40 water-sourced and extra-intestinal genomes from the Touchon data set.

Supplemental Figures msystems.01044-24-s0009.pdf Figures S1 to S6.

ASM does not own the copyrights to Supplemental Material that may be linked to, or accessed through, an article. The authors have granted ASM a non-exclusive, world-wide license to publish the Supplemental Material files. Please contact the corresponding author directly for reuse.
