# Supplementary material for: Semantic search using protein large language models detects class II microcins in bacterial genomes
Source: mSystems. 2024 Sep 18;9(10):e01044-24. doi: 10.1128/msystems.01044-24 (PMC11494933; doi:10.1128/msystems.01044-24)
Supplement: Supplemental Figures — Figures S1 to S6. [file msystems.01044-24-s0009.pdf]

Supplementary Figures

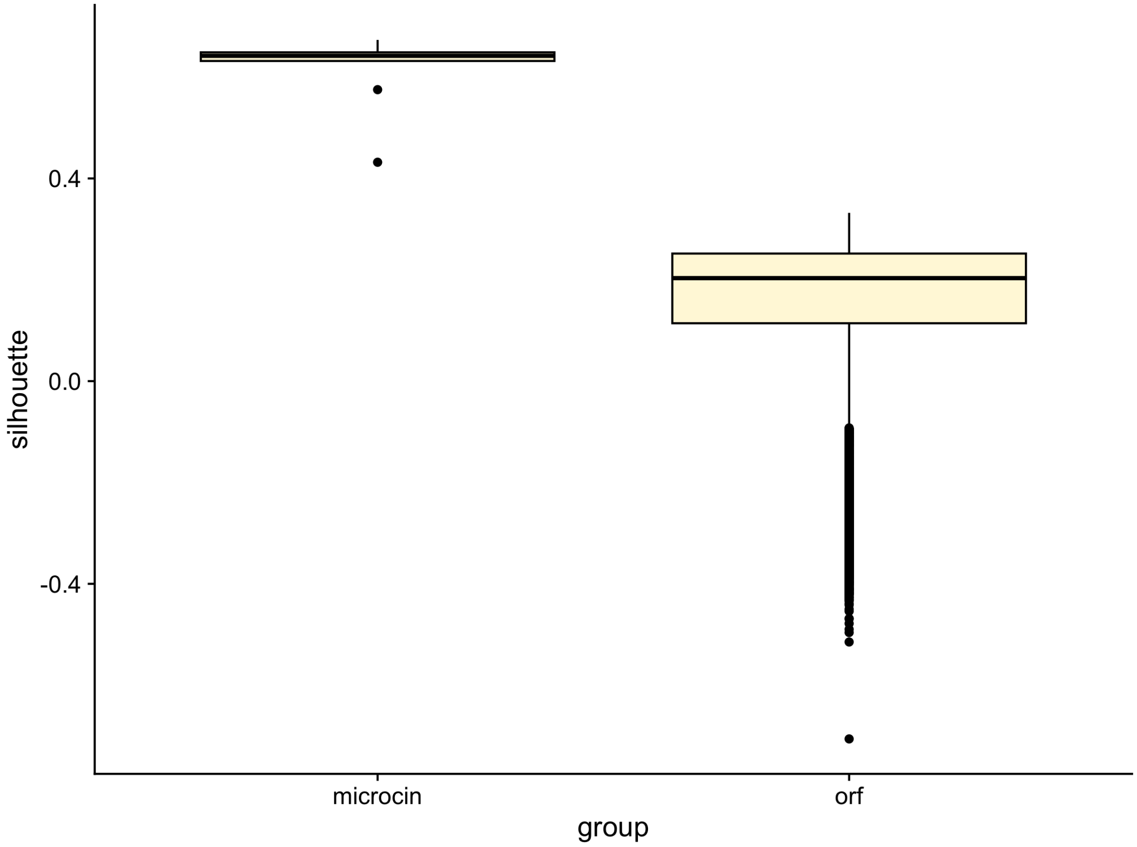

Supplementary Figure S1: Silhouette scores for the microcin and orf embeddings of the *E. coli* 54909 genome. In the orf group, there are 27,745 putative ORFs of microcin length (30–150 amino acids). The microcin group contains the 10 known microcin orfs. The median silhouette score for the microcin group is 0.64, while the median silhouette score for the orf group is 0.20.

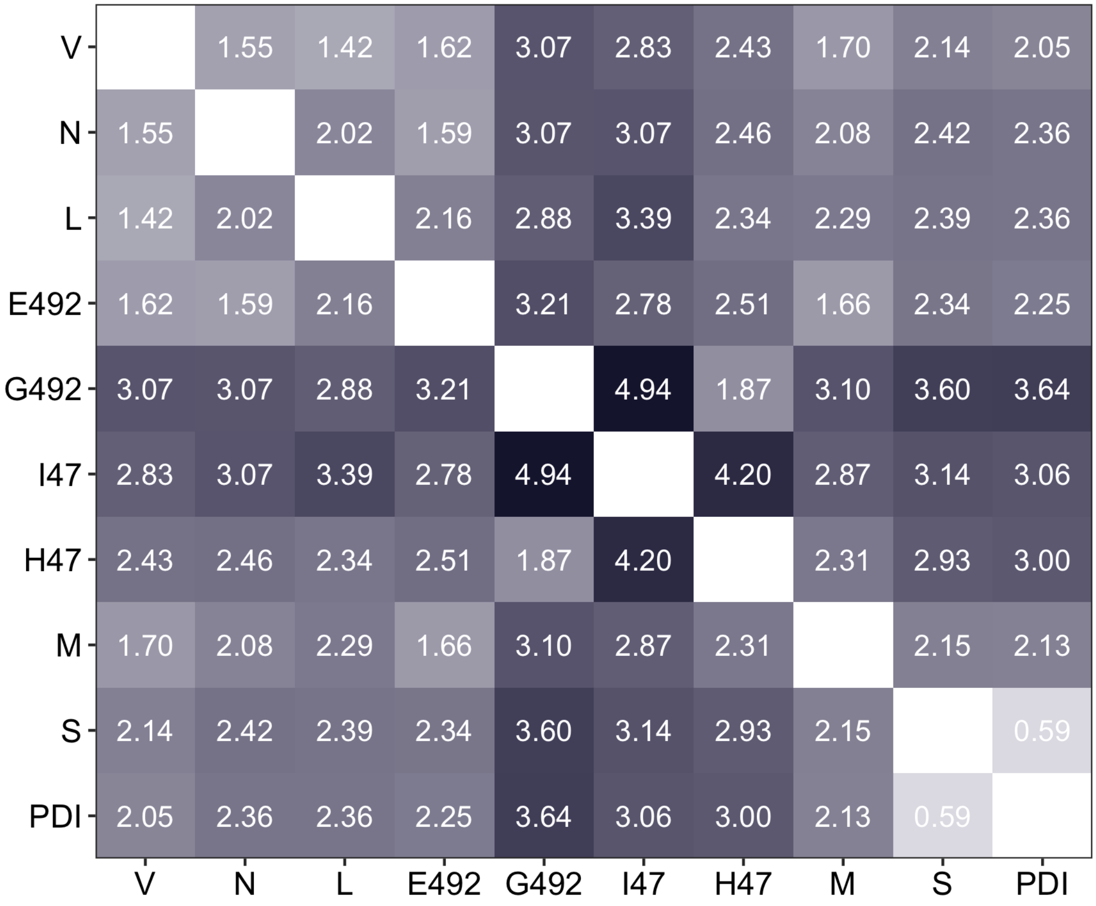

Supplementary Figure S2: Embedding distance between all pairs of the ten known microcins.

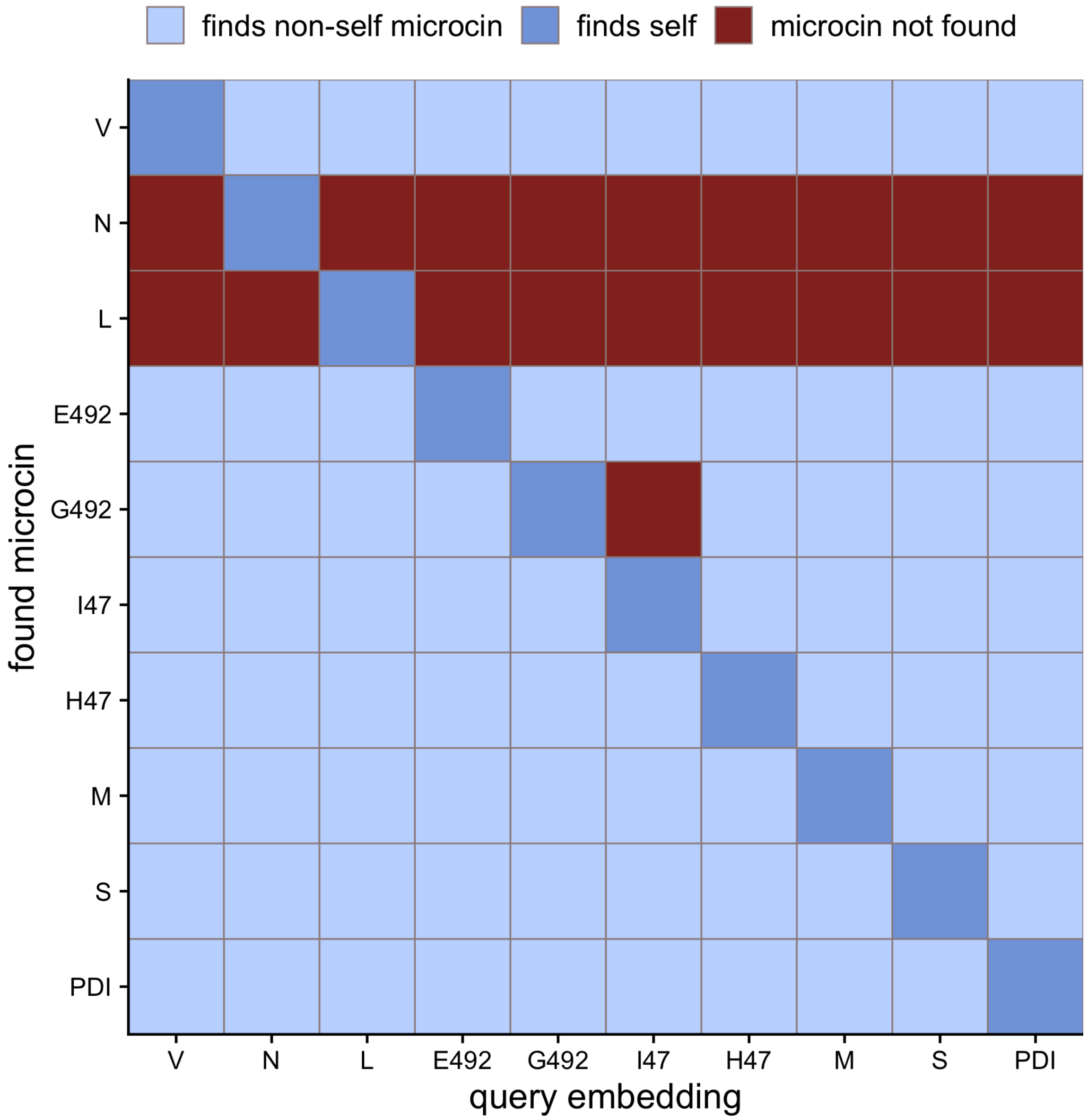

Supplementary Figure S3: Performance of searches via semantic embeddings using the cosine distance. Light blue indicates that a microcin was found using a different microcin as the query. Dark blue indicates that a microcin was found using itself as the query. Red indicates that the microcin was not found.

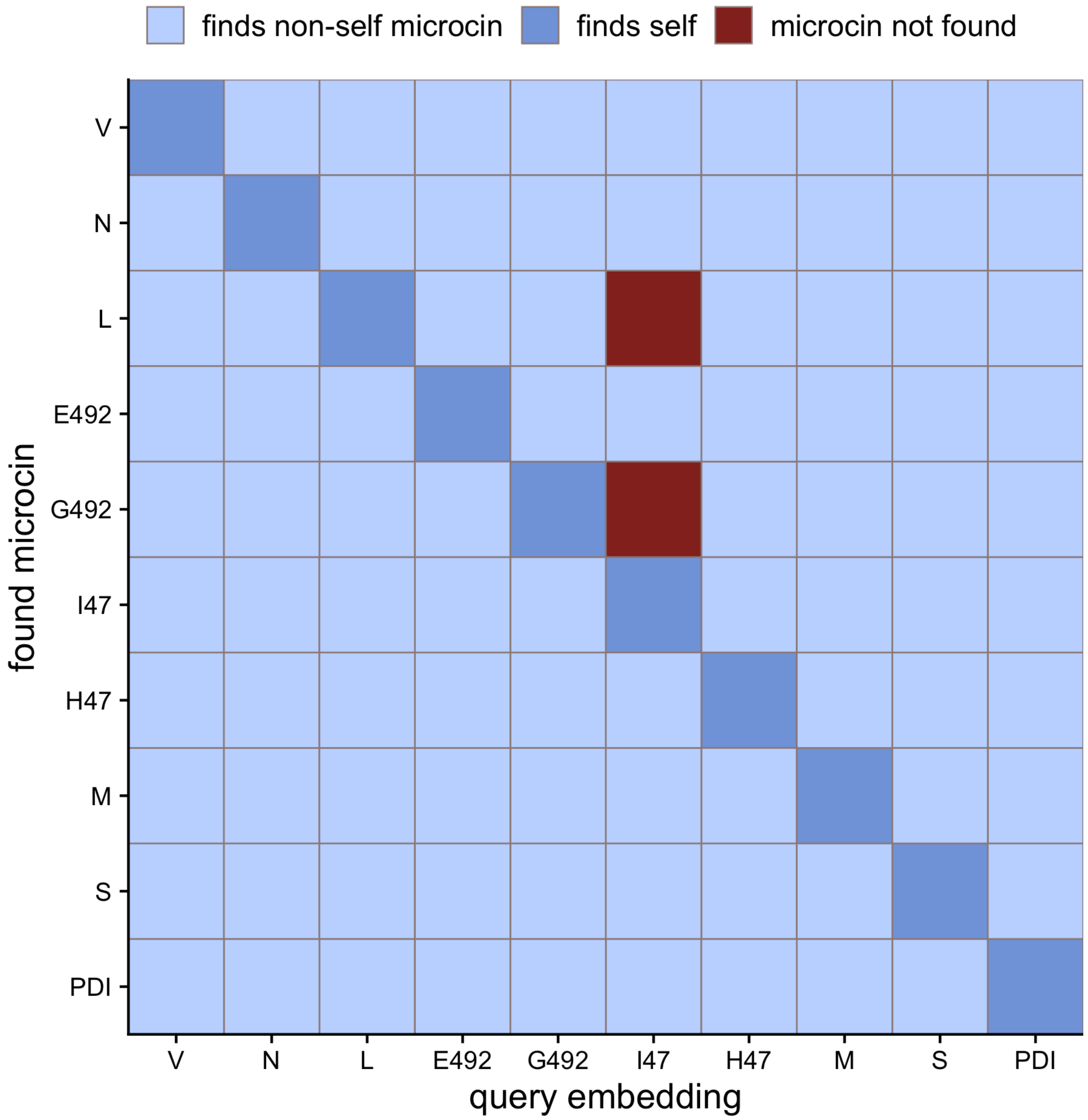

Supplementary Figure S4: Performance of searches via semantic embeddings using the Manhattan distance. Light blue indicates that a microcin was found using a different microcin as the query. Dark blue indicates that a microcin was found using itself as the query. Red indicates that the microcin was not found.

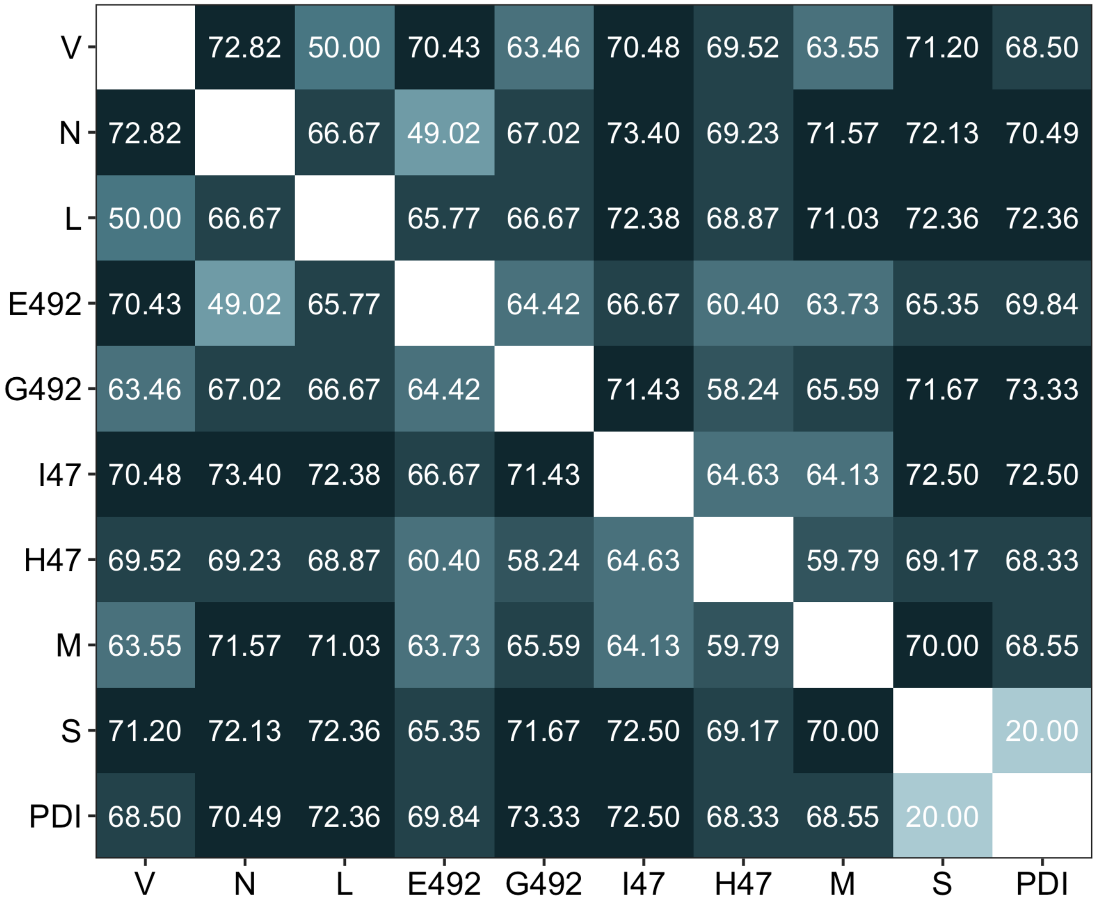

Supplementary Figure S5: Percent sequence divergence between all pairs of the ten known microcins.

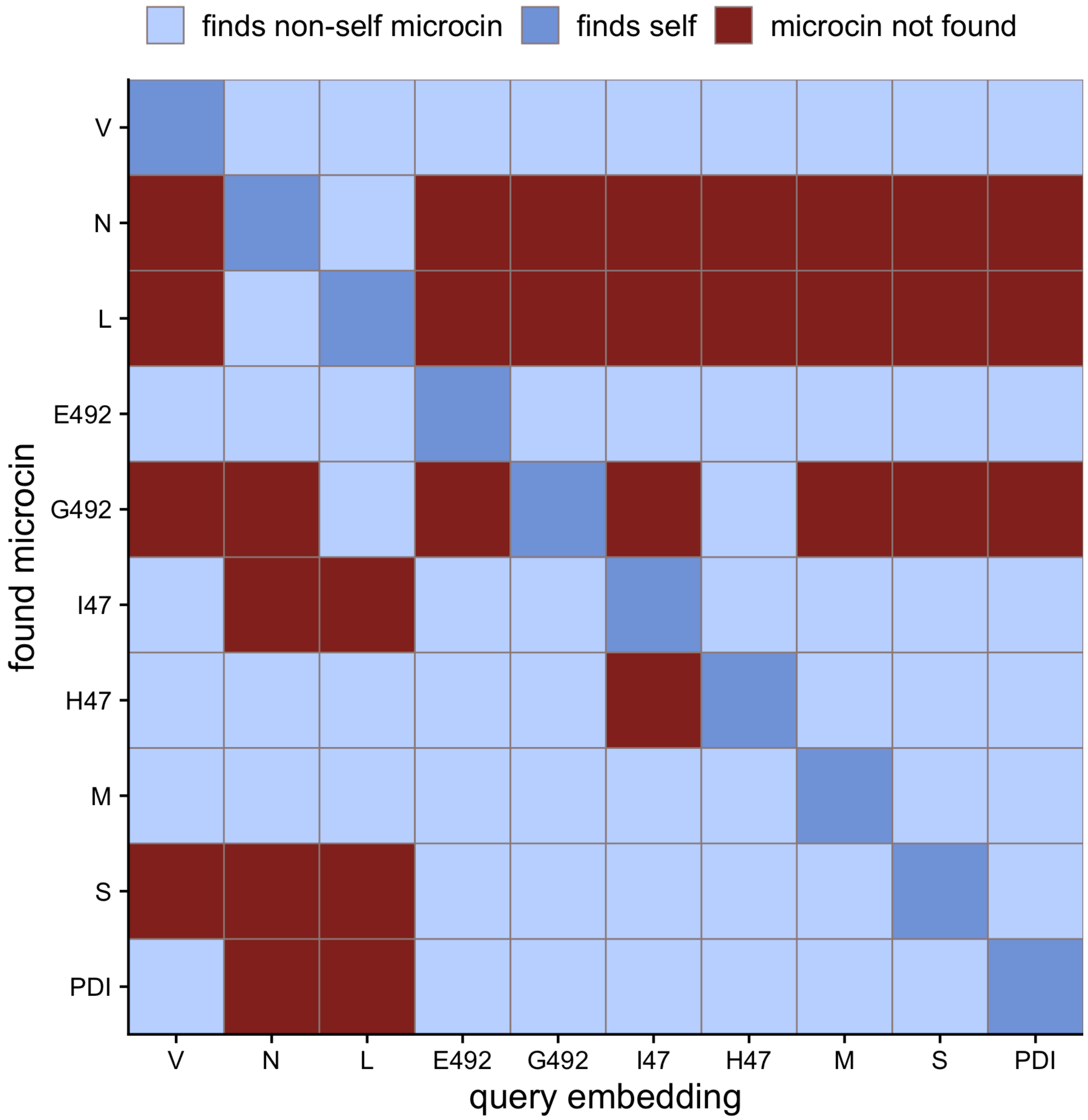

Supplementary Figure S6: Performance of searches via amino acid composition rather than semantic embeddings. Light blue indicates that a microcin was found using a different microcin as the query. Dark blue indicates that a microcin was found using itself as the query. Red indicates that the microcin was not found.

Supplementary Files

Supplementary File S1: Amino acid sequences of the ten known microcins, provided in FASTA format.

Supplementary File S2: Microcin–microcin BLAST hits. Contains the columns `orf_number`, `found_microcin`, `query`, `subject`, `percent_sim`, `alignment_length`, `num_mismatches`, `e.value`, `perc_mismatch`, `group`.

Supplementary File S3: Information about the *E. coli*, *Enterobacter* and *Klebsiella* genomes. Sheet a: Information about the dataset of 25 *E. coli* genomes. Contains the columns `name`, `strain_name`, `accenssion_number`, `strain_category`, `phylogroup`, `Genome_ID`, `microcin_hit_count`, `CvaB_hit_count`. Sheet b: Information about the dataset of *Enterobacter* genomes. Contains the columns `accession`, `ncbi_organism_name`, `cinful_CvaB_found` (indicates whether a CvaB was previously found by cinful—the value is `TRUE` for all genomes in this set), `cinful_microcins_found`. Sheet c: Information about the dataset of *Klebsiella* genomes. Contains the columns `accession`, `ncbi_organism_name`, `cinful_microcins_found` (number of microcins found by cinful), `cinful_CvaB_found` (indicates whether a CvaB was previously found by cinful—the value is `TRUE` for all genomes in this set).

Supplementary File S4: Alignment of embedding hits collected from 25 *E. coli* genomes.

Supplementary File S5: Putative microcins found in the *E. coli*, *Enterobacter*, and *Klebsiella* datasets.

Supplementary File S6: Alignment of embedding hits collected from 44 *Enterobacter* genomes.

Supplementary File S7: Alignment of embedding hits collected from 46 *Klebsiella* genomes.

Supplementary File S8: Cinful results for 40 water-sourced and extra-intestinal genomes from the Touchon dataset. Contains the columns `name`, `strain_name`, `accession_number`, `strain_category` (origin of the bacteria), `phylogroup`, `Genome_ID`, `microcin_hit_count` (how many microcins were detected by Cinful), `CvaB_hit_count` (if Cinful detected a microcin exporter protein), `Assembly` (accession number for retrieval from NCBI).
